# β-Galactosidases from a Sequence-Based Metagenome: Cloning, Expression, Purification and Characterization

**DOI:** 10.3390/microorganisms9010055

**Published:** 2020-12-28

**Authors:** María Florencia Eberhardt, José Matías Irazoqui, Ariel Fernando Amadio

**Affiliations:** Instituto de Investigación de la Cadena Láctea (CONICET-INTA), Rafaela 2300, Argentina; eberhardt.maria@inta.gob.ar (M.F.E.); irazoqui.jose@inta.gob.ar (J.M.I.)

**Keywords:** sequence-based metagenomics, β-galactosidase, stabilization ponds, value-added products

## Abstract

Stabilization ponds are a common treatment technology for wastewater generated by dairy industries. Large proportions of cheese whey are thrown into these ponds, creating an environmental problem because of the large volume produced and the high biological and chemical oxygen demands. Due to its composition, mainly lactose and proteins, it can be considered as a raw material for value-added products, through physicochemical or enzymatic treatments. β-Galactosidases (EC 3.2.1.23) are lactose modifying enzymes that can transform lactose in free monomers, glucose and galactose, or galactooligosacharides. Here, the identification of novel genes encoding β-galactosidases, identified via whole-genome shotgun sequencing of the metagenome of dairy industries stabilization ponds is reported. The genes were selected based on the conservation of catalytic domains, comparing against the CAZy database, and focusing on families with β-galactosidases activity (GH1, GH2 and GH42). A total of 394 candidate genes were found, all belonging to bacterial species. From these candidates, 12 were selected to be cloned and expressed. A total of six enzymes were expressed, and five cleaved efficiently ortho-nitrophenyl-β-galactoside and lactose. The activity levels of one of these novel β-galactosidase was higher than other enzymes reported from functional metagenomics screening and higher than the only enzyme reported from sequence-based metagenomics. A group of novel mesophilic β-galactosidases from diary stabilization ponds’ metagenomes was successfully identified, cloned and expressed. These novel enzymes provide alternatives for the production of value-added products from dairy industries’ by-products.

## 1. Introduction

Whey is the main by-product of dairy industries. It is composed mainly of lactose (4.5–5% *w/v*), proteins (0.6–0.8% *w/v*), lipids (0.4–0.5–5% *w/v*) and mineral salts (8–10% of dried extract). The estimated production of whey worldwide is about 180–190 million tons/year [1]. The high chemical and biological oxygen demands and the large volume of production make whey an important environmental problem.

β-Galactosidase (β-D-galactoside galactohydrolase or lactase; EC 3.2.1.23) is a type of glycoside hydrolase that has important industrial applications, such as lactose hydrolysis and transgalactosylation [2,3]. Several studies have been published in the past decades on the use of β-galactosidases from different sources for lactose hydrolysis [4,5,6]. Commercial enzymes were obtained from a limited number of species, including *Aspergillus*, *Bacillus* and *Kluyveromyces*. 

In recent years, metagenomic strategies have been developed to identify novel enzymes from both cultivable and uncultivable members of microbial communities. This methodology consists in recovering and studying the complete genomic DNA from a particular environment [7]. There are two main metagenomics strategies for bioprospecting new enzymes: sequence-based metagenomics and functional or activity-based metagenomics [8]. Sequence-based approaches involve the sequencing of the whole metagenome DNA, searching for novel homologues by comparing predicted genes with sequences of known activity. These genes are then cloned into expression vectors and expressed in suitable hosts [9]. On the other hand, functional approaches involve the fragmentation of environmental DNA, and the cloning of these fragments into expression vectors. The obtained clones are then functionally screened for specific activities.

In this study, a sequence-based metagenomic analysis of stabilization ponds systems of small dairy industries from the central region of Santa Fe province, Argentina, was performed. Stabilization ponds are the most common treatment technology due to their low operation and maintenance costs [10]. The wastewater generated by the cheese industries are composed mainly of different dilutions of milk (or transformed products, like whey), and washing water containing alkaline and acidic chemicals after the cleaning of bottles, tanks and process equipment (tools and pumps) [11]. Therefore, this kind of environment would be suitable for the proliferation of microbial species with numerous sugar-modifying enzymes.

The present study reports the identification, production and functional characterization of several novel lactose-modifying β-Galactosidases. These novel enzymes are able to use both synthetic and natural substrates, showing better activity values than others previously reported.

## 2. Materials and Methods

### 2.1. Metagenomic Analysis

DNA samples were collected from six stabilization ponds of two small dairy companies in the central region of Santa Fe, named AUR and CYC. DNA was extracted by using PowerWater metagenomic extraction kit (MOBIO) and shotgun sequenced (WGS) by using an Illumina Hiseq1500 platform (rapid run, 2 × 150 bp; INDEAR, Rafaela, Argentina). The samples from each company were assembled by using IDBA_UD [12], and genes were predicted by using Prodigal [13]. Predicted proteins were compared against the CAZy database [14], using HMMer 3.0 [15] and the profiles provided by the dbCAN database [14,16]. To reduce the number of false positives, the alignments covering less than 60% of the profile were discarded.

For the GH2 family, an analysis based on the classification proposed by Talens-Perales et al. [17] was also carried out. First, the seed alignments for each of the core domains present in most classes of GH2 were downloaded from Pfam [17,18]: GH2N (PF02837), GH2d (PF00703), GH2C (PF02836), DUF4981 (PF16353), DUF4982 (PF16355) and Bgal_Small_N (PF02929). Then, the putative GH2 sequences were classified, based on which domains were found. Finally, using the HMMer website (https://www.ebi.ac.uk/Tools/hmmer/), all the candidate GH2 sequences were compared against the Pfam database, to determine if other domains were present in the C-terminal end of each sequence.

Lastly, to guide the selection of candidate sequences to clone and express, each sequence was compared to the nr database from NCBI [19], using BLAST, and used the best hit to assign a taxonomic lineage. The main goal was to select candidates spanning different families and a wide taxonomic range.

### 2.2. Cloning Full-Length β-Galactosidases Genes

*Escherichia coli* DH5α (Thermo fisher, Waltham, MA, USA) was used for plasmid propagation during cloning steps, and *E. coli* BL21(DE3) served as expression host. *E. coli* strains were made chemically competent and transformed according to the protocols described by Sambrook [20]. The concentrations of kanamycin and chloramphenicol used in the study were 50 and 20 mg/L, respectively.

Twelve full-length putative β-galactosidases genes were PCR amplified from the metagenomic DNA, using specific primers (Supplementary Materials Table S1) with proofreading DNA polymerases (AccuPrime, Thermo Scientific, Waltham, MA, USA) and cloned into pGEM-T-easy vector (Promega, Madison, WI, USA). Clone vectors carrying putative β-galactosidases were capillary sequenced for confirmation.

All the expression systems were constructed by using pET-TEV vector, a pET28based vector with a protease TEV recognition site downstream the N-terminal 6-His tag. PCR primers used to amplify complete genes incorporating restriction sites are listed in Supplementary Materials Table S1. PCR products and the pET-TEV vector were digested with defined restriction enzymes and ligated to generate all the expression constructions. Restriction enzymes were obtained from Thermo Scientific (Waltham, MA, USA) and used as recommended.

One enzyme, βgal1, was not produced in the soluble fraction, therefore *Saccharomyces cerevisiae* BJ3505 (Eastman Kodak Company, Rochester, NY, USA) was used as host instead. The β-galactosidase gene was PCR amplified by using primers containing homologous regions to the YEp vector (Eastman Kodak Company). Then, the construction was cloned by yeast recombinases, as reported in Becerra, 2001 [21]. These constructions incorporate a signal peptide that allows the secretion of the protein to media. 

### 2.3. Expression of β-Galactosidases

Recombinant *E. coli* strains were grown overnight at 37 °C in 2 mL of Lysogeny broth (LB) medium. This culture was used to inoculate 10 mL of minimal medium M9 [22], to an initial OD_600nm_ = 0.1. Following incubation at 200 rpm and 37 °C, protein expression was induced at OD_600nm_ = 1 with 100 µM isopropyl β-D-1-galactopyranoside (IPTG). Culture was incubated for 6 additional hours, at 22 °C.

For yeast expression, cells were grown at 30 °C and 250 rpm, for 7 days, in 15 mL of YPHSM medium (1% (*w/v*) glucose, 3% (*v/v*) glycerol, 1% (*w/v*) yeast extract and 8% (*w/v*) peptone) to recover proteins in supernatant fraction.

### 2.4. Enzyme Purification

Cells and supernatants of the cultures were separated by centrifugation for 15 min at 5000 rpm. Cell pellets were resuspended in phosphate buffer (100 mM phosphate pH 8.0, 300 mM NaCl). Resuspended cells were disrupted on ice by sonication until clarification. After cell disruption, crude extracts were clarified by centrifugation for 15 min, at 15,000 rpm.

Enzymes were purified by affinity chromatography, using Ni^2+^-NTA Agarose resin (Invitrogen, Carlsbad, CA, USA) according to the protocol supplied by the manufacturer. Crude extracts and purified enzymes were analyzed by SDS-PAGE on 12% gels [23]. Protein concentration of purified enzymes was measured in Nanodrop (Thermo Scientific).

### 2.5. Characterization of β-Galactosidases 

Enzymatic activity was measured by using 2-nitrophenyl-β-D-galactopyranoside (ONPG). Purified proteins were diluted in buffer Z (100 mM Na_2_HPO_4_, 40 mM NaH_2_PO_4_, 10 mM KCl and 1.6 mM MgSO_4_). After incubation at 30 °C for 4 min, the reaction was started by adding an equal volume of substrate (4 mg/mL) in buffer Z to the enzyme solution. Aliquots of the reaction mixture were extracted and the reaction was stopped by adding an equal volume of 1 M Na_2_CO_3_. Released p-nitrophenol was measured by UV absorbance at 405 nm. β-Galactosidase activity is expressed in enzymatic units (U) and defined as the amount of enzyme capable of releasing 1 μmol of product (p-nitrophenol) per min (i.e., μmol min^−1^ mL^−1^) under the experimental conditions.

The pH dependence of enzymatic activity was evaluated by the standard β-galactosidase assay with ONPG at pH ranging from 4 to 10, using Briton–Robinson buffer (20 mM acetic acid, 20 mM phosphoric acid and 20 mM boric acid titrated with 1 M NaOH to the desired pH). The optimum temperature for the hydrolytic activity with ONPG was evaluated between 30 and 65 °C. The thermostability was evaluated by incubating the pure enzyme in buffer Z, at optimal temperature, for 24 h. The residual activities were measured regularly with ONPG as substrate. To study the effect of different ions (MgCl_2_, ZnSO_4_, CaCl_2_ and KCl) on enzyme activity, assays were carried out, adding different concentration (1 o 10 mM) of salts to the standard β-galactosidase assay with ONPG.

### 2.6. Evaluation of Hydrolytic and Transgalactolytic Activities

Lactose hydrolysis was measured by the glucose production. Purified enzymes were diluted in buffer Z. The reaction time was 20–960 min at optimum temperature. The reaction was stopped by heating at 96 °C for 5 min. β-Galactosidase activity is expressed in enzymatic units (U), defined as the amount of enzyme capable of liberating 1 μmol of product (D-glucose) per min, under the experimental conditions (i.e., μmol min^−1^ mL^−1^). Glucose concentration was measured by using the commercial kit D-Glucose GOD-POD (Wiener).

GOS concentrations were determined by HPLC (HPLC Waters Breeze I). Sugar Pack Waters column (6.5 mm × 300 mm) and 100 μM EDTA-Calcium (Sigma Aldrich, St. Louis, MO, USA) were used as the mobile phase (column temperature, 80 °C; sensor temperature, 37 °C; sensitivity, 32; flow, 0.5 mL/min). Eluted sugars were detected with a Waters 2414 refractive-index detector. The identification and quantification of sugars by HPLC was done by using a mixture of stachyose, raffinose, sucrose and galactose as standards.

### 2.7. Kinetics of Hydrolysis

Purified recombinant proteins were used to the characterization of kinetic parameters. β-Galactosidase activity was assayed (as described above) at different substrate concentrations (0–20 mM). The reaction times were 6–20 min at 30 °C. Measurements were made in triplicate.

## 3. Results

### 3.1. CAZymes Prediction

A total of 2,134,079 ORFs were predicted from the two metagenomic assemblies, from which 21,347 were classified as CAZymes (Table 1 and Supplementary Materials Table S2). There were 221 different families identified in the five main classes of enzymes defined by CAZy. While the most abundant class of CAZymes was the glycoside transferases (GT), with 10,028 candidates across 66 families, the glycoside hydrolases (GHs) was the most diverse class observed (107 families with 8126 candidates).

When compared, the two datasets showed similar diversity, sharing 159 of the 221 families identified (>70%). For the GHs, which are the main focus of this study, from the 107 different families, 86 (>80%) were present in both datasets. In the CYC dataset, there were 14 families found that were absent in AUR, but the number of genes on each one was very low, between one and three hits. Likewise, seven families were found only in the AUR dataset, but again the hit count was really low, with one or two hits per family.

Of all the families of interest, GH2 was the most abundant, with 183 candidates, closely followed by GH1, with 142 putative sequences. Lastly, for the family GH42, 54 candidates were identified.

### 3.2. Taxonomic Classification

**Figure 1:**
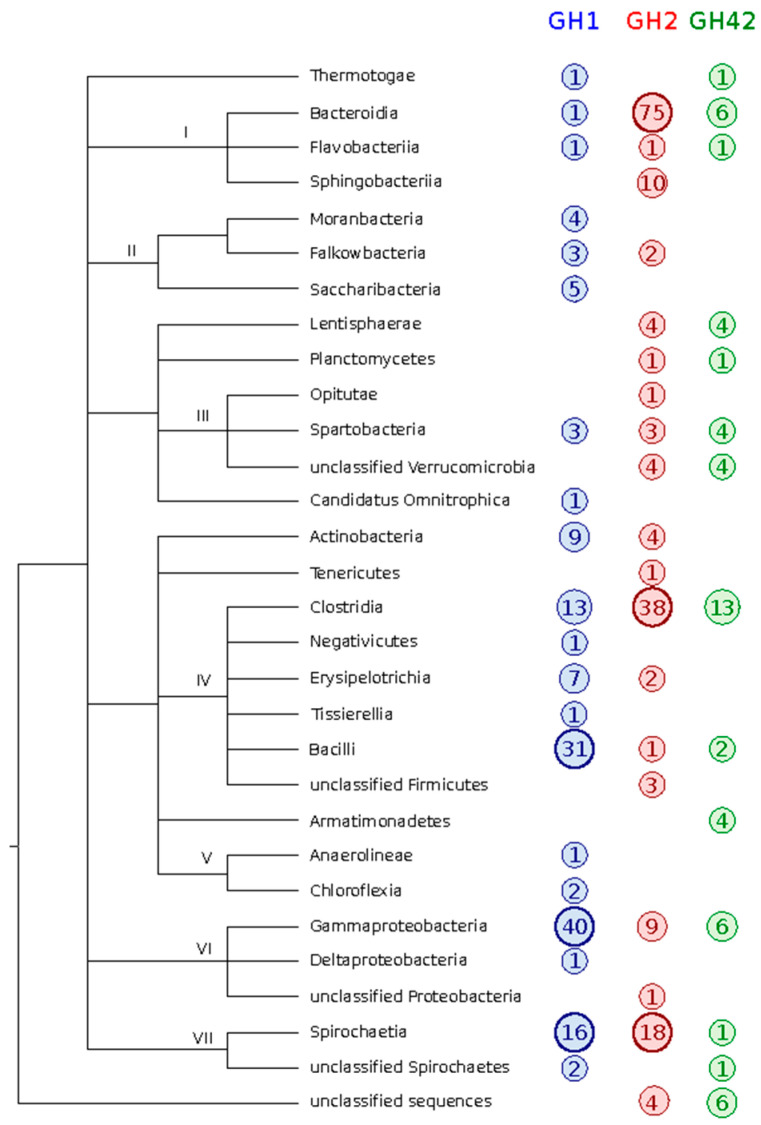
Taxonomic classification of the GH found. All the candidates GH were classified to the class level, using the best hit of BLAST against the nr database. I, *Bacteroidetes*; II, *Patescibacteria*; III, *Verrucomicrobia*; IV, *Firmicutes*; V, *Chloroflexi*; VI, *Proteobacteria*; VII, *Spirochaetes*.

Most of the identified sequences had a hit in the nr database of GenBank and, therefore, could be taxonomically classified, except for 10 candidates that did not have any match and remained as “unclassified” (Figure 1 and Supplementary Materials Table S3). The GH found were distributed among 14 different phyla, although more than 50% of them belong to the *Firmicutes* and *Bacteroidetes* phyla. GH42 was the most evenly distributed family, with 5 different phyla being predominant. However, for the other two families, the distribution is more skewed. For GH1 over 65% of the candidates are distributed between two phyla, namely *Firmicutes* (37.3%) and *Proteobacteria* (28.8%), while for GH2, *Bacteroidetes* (47.5%) and *Firmicutes* (24%) are the most represented taxa. Interestingly, even though *Bacteroidetes* are one of the most abundant phyla, almost all the sequences are GH2 (92%).

### 3.3. Glycoside Hydrolases 2 Classification

Talens-Perales et al. [17] described five different domain architectures (DA) for GH2, from which three were reported to have β-galactosidase activity: types II, III and V. They also reported that two domains were present in every DA, a sugar-binding domain in the N-terminal end (PF02837) and a TIM barrel domain (PF02836); the combination of the other four domains defined the DA type. 

From the original 183 sequences detected, only 79 (43%) were classified in any of the proposed subgroups (Supplementary Materials Table S4). Type III was the most abundant class found, with 39 sequences. For class V, another of the β-galactosidases classes, 12 sequences were found, while for type II, no candidates were found. Interestingly, six sequences showed a DA not reported by Talens-Perales et al. They had the GH2N, GH2d, GH2C, and DUF4981 domains, like DA type 3, but lacked the C-terminal “Bgal-Small-N” domain.

### 3.4. Gene Selection, Design and Expression Vector Construction

The sequences selected for cloning and expression in heterologous systems are listed in Table 2. The selection was made to capture both family and taxonomic diversity. Ten of the twelve selected enzymes were successfully amplified from the metagenomic DNA (Table 2). Confirmed gene sequences were again PCR amplified with oligonucleotides incorporating restriction sites (Supplementary Materials Table S1) to clone in pET-TEV expression vectors. 

### 3.5. Gene Expression, Enzyme Purification and Activity Assays

**Figure 2:**
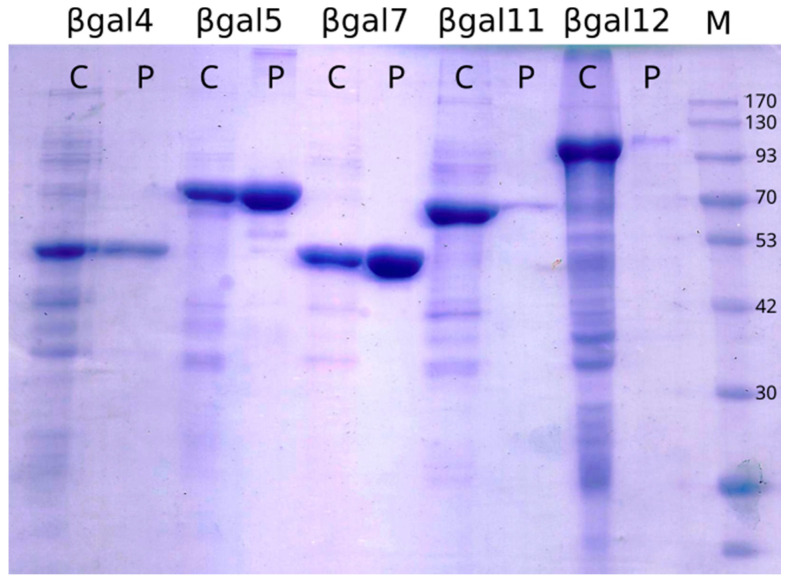
SDS-PAGE of β-galactosidases expression. Crude extracts (C) and affinity purified (P) samples. M, molecular weight marker.

The analysis of soluble and insoluble fractions of cell lysates by SDS-PAGE showed that six of the selected genes were successfully expressed. The molecular weight observed from the accumulation in the total cell fraction agreed with the predicted weights (Figure 2 and Supplementary Materials Figure S1). Soluble purified proteins were obtained to assess activity for the six enzymes produced.

### 3.6. Purification of the Novel β-Galactosidases

All individual proteins were obtained from 25 mL culture and purified passing cell lysates or supernatant (for βgal1) through aa Ni^2+^-NTA affinity resin. The quantification of pure proteins is listed in Table 3. βgal1, βgal5 and βgal7 showed that the highest over-expressions, in consequence, produced higher amounts of pure proteins. For βgal11 and βgal12, more than a half of the over-expressed proteins accumulated at the insoluble fraction (data not shown), producing lower pure protein yield.

Purified enzymes were used for activity assays with chromogenic substrate ONPG (Sigma). In that way, β-galactosidase activity was confirmed in five of six purified proteins. Specific activities and some enzyme properties are shown in Table 3. The most significant specific activity was obtained from βgal5.

### 3.7. Determination of Optimal Reaction Conditions

**Figure 3:**
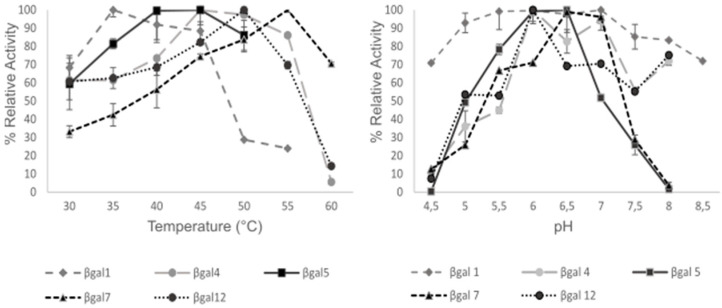
Optimal pH and temperature for β-galactosidases. Enzyme activities were measured by using ONPG as the substrate. The higher activity value for each enzyme was considered as 100%. Data were obtained from biological replicates (*n* = 3); the error bars indicate standard deviation.

Enzyme activity was also evaluated by using purified recombinant β-galactosidases at pH ranging from 4 to 8, using ONPG substrate. Optimal temperatures were determined in the range of 30 to 60 °C with the same substrate. Results are shown in Figure 3 and Table 3. Enzyme βgal1 shows stability at a wide range of acidic pH, between 5.5 to 7. This enzyme is mesophilic, with optimal temperature at 35 °C. βgal5, βgal4 and βgal7, have slightly acidic optimal pH, but they show highest activities at high temperatures, from 45 to 55 °C. βgal12, has also high activity at high temperature, 50 °C, and its optimal pH is a little bit acidophilic, around 6.

### 3.8. Lactose Hydrolysis, Transglycosylation Activity and Kinetic Parameters

Those β-galactosidases that were active with the synthetic substrate were tested with natural substrate, lactose. Reaction mixture is composed of 160 mM lactose dissolved in buffer Z and the reaction took place at optimal temperature for 20 min and up to 8 h. As was seen with ONPG, the best specific activity was again obtained by βgal5 (Table 4).

**Figure 4:**
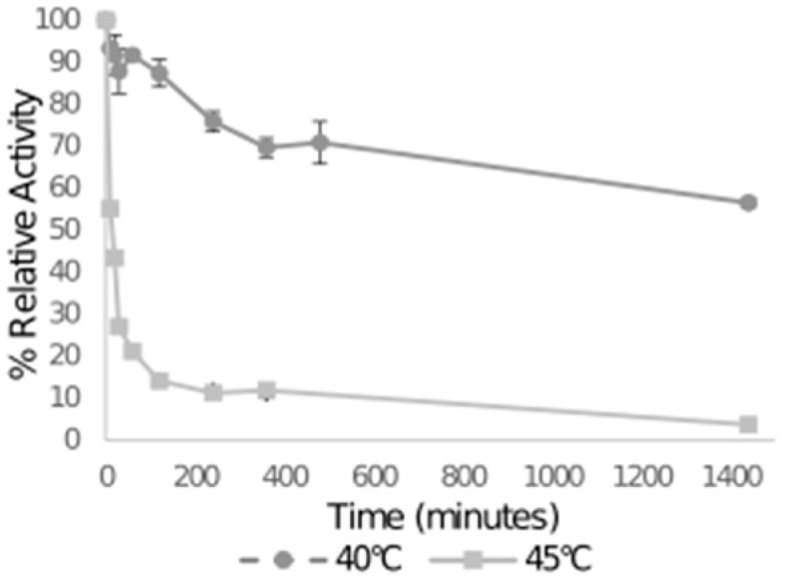
Thermostability assay. Enzyme activities were measured by using ONPG as the substrate. The higher activity value for each enzyme was considered as 100%. Data were obtained from biological replicates (*n* = 3); the error bar indicates standard deviation.

The enzyme βgal5 was characterized, because its activity was significantly higher than the rest of the enzyme candidates. First, thermostability was tested. As it shows in Figure 4, at the optimal temperature of 40 °C, the enzyme activity remains over 85% after an hour incubation, and still over 55% after 8 h. However, at 45 °C, only 20% of the enzyme activity was retained after 1 h incubation. This result shows that βgal5 was unstable at high temperatures.

The effects of four cations on the activity of βgal5 were also studied (Table 5). The activity was increased by 26% after the addition of Ca^2+^, 12% with Zn^2+^ and 10% with K^+^, compared to the control. The activity of βgal5 was inhibited by the addition of Mg^2+^ (14%), compared to its activity without cations (100%).

The substrate specificity of βgal5 was determined by using seven different substrates. The enzyme preferred 4-nitrophenyl-β-D-glucopyranoside as its substrate and exhibited some activity, using 4-nitrophenyl-β-D-fucopyranoside. Meanwhile, it showed no activity with 4-nitrophenyl-α-D-xylopyranoside, 4-nitrophenyl-β-D-xylopyranoside, 4-nitrophenyl-α-D-mannopyranoside, 4-nitrophenyl-α-D-glucopyranoside or 4-nitrophenyl-β-D-mannopyranoside.

Kinetic parameters of βgal5 were calculated by using different concentrations (2.5–20 mM) of ONPG, and the values obtained were as follows: Vmax = 93, 46 µM/min, and Km = 14, 55 mM (Supplementary Materials Figure S2). When lactose was used, it resulted in Vmax = 18, 33 µM/min, and Km = 400 mM (Supplementary Materials Figure S2). 

Transgalactosylation activity of βgal5 was investigated using HPLC analyses, with lactose as the substrate and acceptor. The results showed that the capability of synthesizing GOSs from lactose is very low. HPLC showed that, at optimal conditions, 40 °C and pH 5.5, with a 40% of initial lactose concentration, βgal5 was able to transform around 2% of lactose into a trisaccharide after 8 h of reaction. 

## 4. Discussion

In recent years, with the improvement of sequencing technologies, screening uncultured microorganisms by sequence-based comparison to known sequences has been a useful approach for the identification of novel enzymes with potential for industrial applications [24,25,26].

To the best of our knowledge, this is the first metagenome from wastewater stabilization of dairy ponds. Due to the composition of the wastewater, containing mainly dilutions of milk (or transformed products, like whey), and washing water, the microorganisms in this environment potentially have different kinds of carbohydrates-modifying enzymes. Our results showed that around 1 out of 100 ORFs is a CAZyme, and around 1 out of 50 CAZymes is a putative β-galactosidase. Our focus was on three GH families, GH1, GH2 and GH42, trying to identify enzymes from a wide taxonomic range. A total of 379 candidates were identified. When compared to the nr database, most sequences matched against bacterial entries, including 14 different phyla, but 10 sequences did not have any close match (at least 50% identity); thus, they remained unclassified.

The identification of the enzymes was based on the presence of catalytic domains, as described in the dbCAN database. However, the enormous diversity in the enzyme families studied makes the selection process even more complex (according to CAZy, GH1 family has over 20 different activities reported and the GH2 family, 10). The GH2 family has been extensively studied, and it has been suggested that different activities may be linked to the combination of different small domains. Using these criteria, the number of positive hits for the GH2 family was limited from 183 to 79 (43%). As the number of well-characterized enzymes for other families increases, similar effort to identify key domains for each activity could lead to the more precise identification of novel enzymes.

Based on information of their taxonomy and the GH family they belonged to, 12 candidate genes were selected, from which six proteins were efficiently expressed, and five of them were active, using both synthetic and natural substrates. This represents a 40% success in the rate of expression. All of those enzymes cleaved efficiently the ONPG and lactose at physiological pH (5–7), with optimal temperatures between 30 and 55 °C. Since the pH of natural milk is about 6.7–6.8, an ideal β-galactosidase for lactose hydrolysis in milk should be optimally in this range. βgal1, βgal5 and βgal7 displayed a more suitable optimum pH for lactose hydrolysis in milk.

The highest specific activity on ONPG and lactose was observed by using βgal5, at 40 °C (280 and 142 U/mg, respectively). Specific activity on ONPG is twice as high as the specific activity on the natural substrate, lactose. This has been observed for several β-galactosidases of the GH42 family: a thermostable metagenome-based Gal308 [27], cold-adapted metagenome-derived ZD410 [28], thermostable β-galactosidase from *C. saccharolyticus* [29] and a β-galactosidase from *Alicyclobacillus acidocaldarius* [30]. 

One of the limitations of the sequence-based screening is the selection of candidates to work with in the wet lab. Due to the high number of candidates that can be identified in silico, it is not feasible to test them all by using the followed strategy. Moreover, as stated before, the functional diversity in these enzymes families adds an extra layer of complexity. To the best of our knowledge, only one screening from a sequence-based metagenome led to finding an alkaline βgal375 that is able to synthesize galactooligosaccharides [31]. The specific activity on chromogenic (PNPG) and natural (lactose) substrates was 15.6 and 0.96 U/mg, respectively, and transglycosylation activity is 73.36 g/L of GOS. The specific activity levels of the enzyme βgal5, presented in this study, were three orders of magnitude higher, using lactose as substrate, making it an interesting candidate for lactose transformation (Table 6).

Using a functional metagenomic approach, mostly soil metagenome derived β-galactosidases were described. A cold-adapted ZD410 [28] that is able to hydrolyze lactose from milk at 4 °C, the thermostable Gal308 [27] with a high enzymatic activity (47.6 U/mg) and M1 [32] which is able to completely hydrolyzed milk lactose in 25 h (>99.9% conversion), and Lac161_ORF7 [33] represent a novel β-galactosidase family. The enzyme BGal17E2 [34] is a cold-adapted, alkaline β-galactosidase identified from submarine ikaite columns. None of these enzymes showed higher levels of activity than βgal5 (Table 6). No other enzymes were reported along with ZD410 and Gal308 in their respective publications; meanwhile, M1 was one of six candidates featuring promising properties, as compared to the commercial reference. The biggest limitation of the functional approach is the efficiency of screening. The incidence rate of positive clones when performing a native screen in the environmental clone libraries is 1:31,190 for glycosidases [35]. There are multiple factors that affect the screening success, such as the environment studied, DNA extraction method, enzyme activity, the substrates used, the abundance of corresponding genes, cloning vector, expression system or host cells and screening conditions [35]. The selection of the appropriate substrate is crucial, considering that the substrate azurine hydroxyethyl cellulose, a unique substrate for the measurement of endo-cellulase, provided a major incidence rate (1:108) (93 out of a total of 10,000 clones tested) [35,36]. In contrast, 5- bromo-4-chloro-3-indolyl-β-d-galactopyranoside (X-gal), a common substrate for the screening of β-galactosidase activity at high frequency, is the substrate providing in some cases the lowest number of positive hits (1:700,000) [37]. The functional metagenomic approach has the advantage that any positive result represents a novel functional enzyme, but given its low efficiency and the high number of variables that could affect the expression of a gene, the sequence-based method followed in this work proved to be an efficient approach for the identification of novel enzymes. Taking into account the continuous drop in the price of sequencing, these approaches are more common. A proper bioinformatic identification and selection processes can lead to an acceptable or even better rate of success in the production of enzymes of a desired activity.

In conclusion, we successfully identified, cloned and expressed a group of five novel mesophilic β-galactosidases from diary stabilization ponds’ metagenomes. All enzymes are active with both synthetic and natural substrates. Further optimizations of these enzymes could lead to an increased specific activity with lactose or maximize the hydrolysis or transgalactosylation activity and provide a range of enzymatic activities applicable to industrial processes.

## Figures and Tables

**Table 1:** Prediction of CAZymes in the metagenomic samples. Classification of the putative CAZymes found in each sample. GH, glycoside hydrolase; GT, glycoside transferase; PL, polysaccharide lyase; CE, carbohydrate esterase; AA, auxiliary activities.

	AUR		CYC		Total	
Type	Genes	Families	Genes	Families	Genes	Families
GH	2970	93	5156	100	8126	107
GT	3896	46	6132	60	10,028	66
PL	104	13	236	17	340	17
CE	940	15	1762	15	2702	15
AA	69	5	82	6	151	6

**Table 2:** Putative β-galactosidases selected from metagenomes. Enzymes noted with * could not be amplified by PCR. MM, molecular mass.

**Enzyme**	**Gene Name**	**Length (aa)**	**MM (kDA)**	**Family**	**Lineage**
βgal1	AUR.contig-100_513_12	453	51.64	GH1	*Verrucomicrobia*
βgal2 *	AUR.contig-100_17147_1	449	49.94	GH1	*Chloroflexi*
βgal3 *	AUR.contig-100_3427_6	477	51.99	GH1	*Actinobacteria*
βgal4	AUR.contig-100_2450_8	468	54.15	GH1	*Firmicutes*
βgal5	AUR.contig-100_1620_7	677	77.67	GH42	*Firmicutes*
βgal6	AUR.contig-100_1130_5	619	71.33	GH2	*Bacteroidetes*
βgal7	CYC.contig-100_2881_9	469	52.92	GH1	*Proteobacteria*
βgal8	CYC.contig-100_4237_11	698	80.42	GH42	*Bacteroidetes*
βgal9	CYC.contig-100_9789_2	480	54.69	GH1	*Firmicutes*
βgal10	CYC.contig-100_1083_6	605	67.99	GH2	*Proteobacteria*
βgal11	CYC.contig-100_2840_6	670	76.14	GH42	*Proteobacteria*
βgal12	AUR.contig-100_220_22	1036	117.69	GH2	*Verrucomicrobia*

**Table 3:** Specific activity on 2-nitrophenyl-β-D-galactopyranoside (ONPG) and β-galactosidases properties. Data were obtained from biological replicates (*n* = 3). MM, molecular mass. ND, not detected.

Enzyme	Enzyme Activity (U/mg)	GH Family	Optimum pH	Optimum Temperature	Pure Protein Concentration (mg/mL)
βgal1	4.65 ± 0.17	GH1	6.5	35 °C	1.550
βgal4	4.10 ± 0.08	GH1	6.0	45 °C	0.225
βgal5	294.9 ± 19.06	GH42	6.5	40 °C	1.470
βgal7	4.49 ± 0.06	GH1	6.5	55 °C	2.030
βgal7	ND	GH42	-	-	0.180
βgal12	5.71 ± 0.13	GH2	6.0	50 °C	0.135

**Table 4:** β-Galactosidase activity on lactose. Data were obtained from biological replicates (*n* = 3).

Enzyme	Lactose Activity (U/mg)
βgal1	8.23 ± 0.08
βgal4	0.96 ± 0.02
βgal5	142.33 ± 18.67
βgal7	0.50 ± 0.01
βgal12	2.34 ± 0.22

**Table 5:** Effect of metal ions on βgal5 activity. Data were obtained from biological replicates (*n* = 3).

Reagent Added	Concentration (mM)	Relative Activity (%)
MgCl_2_	10	86.4 ± 7.7
	1	99.0 ± 3.0
ZnSO_4_	10	108.1 ± 4.4
	1	111.9 ± 7.4
CaCl_2_	10	121.1 ± 3.7
	1	126.6 ± 3.5
KCl	10	102.1 ± 3.2
	1	109.7 ± 10.9

**Table 6:** Comparison of metagenome-derived β-galactosidases. The pH and temperature (Temp.) show the optimal reported values. MM, molecular mass in kDa. ND, no data available.

Enzyme	Source	Assay Type	GH Family	MM	Km (mM)		Specific Activity (U/mg)		pH	Temp. (°C)	Reference
					ONPG	Lactose	ONPG	Lactose			
βgal 5	Dairy stabilization ponds	Sequence-based	42	74.5	14.55	403	294.9	142.3	6.5	40	This study
Gal308	Soil	Functional screening	42	95	2.7	7.1	185	47.6	6.8	78	[28]
zd410	Soil	Functional screening	42	78.6	1.7	12.2	243	25.4	7	38	[29]
BGal_375	Soil	Sequence-based	2	112	1.65	ND	15.6	0.96	8	50	[32]
M1	Soil	Functional screening	2	120	ND	14.3	ND	ND	7	37	[33]
BGalI17E2	Submarine ikaite columns	Functional screening	1	52.7	ND	ND	ND	ND	6	37	[34]
Lac161_ORF7	Soil	Functional screening	2	ND	ND	1.8	ND	ND	6	50	[35]

## Supplementary Materials

The following are available online at https://www.mdpi.com/2076-2607/9/1/55/s1. Table S1: Primers used for amplification and cloning. Table S2: Complete list of CAZymes identified in the metagenomes. Table S3: GH taxonomic classification. Table S4: GH subgroup classification. Figure S1: Time course SDS-PAGE analysis of βgal1 expression in *S. cerevisiae*. Figure S2: Kinetic characterization of the enzymatic activity of βgal5.
